# Relevance of anamnesis and of biomarkers in the assessment of smoking among patients with airway disease

**DOI:** 10.1590/S1806-37132015000200001

**Published:** 2015

**Authors:** Ubiratan de Paula Santos

**Affiliations:** 1 Attending Physician. Smoking Cessation Outpatient Clinic, Pulmonary Division, Heart Institute, University of São Paulo, School of Medicine, Hospital das Clínicas, São Paulo, Brazil

"The prevalence of smoking in patients with chronic obstructive pulmonary disease or asthma is high-the relevance of anamnesis and of the use of biomarkers in clinical practice." - Ubiratan de Paula Santos.

Smoking is the leading preventable risk factor for death. It is estimated to have been responsible for 6.3 million deaths globally in 2010. ^(^ 1 ^)^ Studies have suggested that smokers live, on average, 10 years shorter than nonsmokers and that one in every two smokers will die from a tobacco-related disease.^(^ 2 ^,^ 3 ^)^ The reason for such an impact is the presence, in the smoke inhaled from burning tobacco, of approximately 5,300 chemicals, including 250 toxic components, 72 carcinogens (600 trillion molecules of carcinogens per cigarette smoked), and 4 × 10^9^ fine particles/cm³ of inhaled smoke.^(^ 4 ^)^ Among the major impairments associated with smoking are airway and interstitial respiratory diseases, especially COPD and more than 10 types of cancer, including head and neck cancer^(^ 5 ^)^ and lung cancer, which is ranked 1st in the global ranking of cancer deaths,^(^ 6 ^)^ smoking being responsible for more than 80% of cases. In addition to these known morbidities, evidence from new studies suggest that smoking increases the risk of death from renal failure, intestinal ischemia, breast cancer, and prostate cancer,^(^ 7 ^)^ thereby broadening the spectrum and dimension of the effects of smoking. In other diseases such as asthma, although various studies have suggested that smoking is a causal factor, there is still controversy over it; however, evidence confirms that smoking or exposure to environmental tobacco smoke makes asthma control difficult and makes exacerbations more frequent.^(^ 8 ^)^ Because of greater knowledge of the risks and because of the measures adopted by countries, the prevalence of smokers has been gradually declining, especially in Brazil, with a reduction in the prevalence of smokers, in the population aged 18 years or older, from 35.4% to 16.8% between 1989 and 2010.^(^ 9 ^)^

In this issue, the Brazilian Journal of Pulmonology publishes an interesting study^(^ 10 ^)^ involving patients with COPD or asthma and a control group of healthy smokers and nonsmokers, comparing smoking markers to determine the accuracy of the self-report of smoking status. The data are impressive: 29% of the patients with asthma or COPD who self-reported being nonsmokers had high urinary cotinine values and high exhaled CO values. If we consider urinary cotinine levels alone, whose cut-off value of 200 ng/mL is used in order to discriminate smokers from nonsmokers, they were too high to be explained only by the exposure to environmental tobacco smoke, and the prevalence of self-reported nonsmokers who had high levels of this marker reached 38% (29% and 47% of the patients with asthma and COPD, respectively). As indicated in the article,^(^ 10 ^)^ the values observed for false information were higher than those observed in other studies, which can be explained, in part, by differences in the prevalence of smokers in the local populations during the various study periods. Research conducted in various countries has shown that the prevalence of smokers among patients with COPD^(^ 11 ^,^ 12 ^)^ or asthma^(^ 12 ^,^ 13 ^)^ is similar to that observed among smokers in general. In the study by Stelmach et al.,^(^ 10 ^)^ since it was carried out at a specialized care hospital, we should also consider the possibility of patients fearing discontinuation of follow-up as one of the factors influencing such a large number of false reports.

The study^(^ 10 ^)^ results strongly suggest that, in the approach to patients with asthma or COPD, the question on smoking status be reiterated and this status be assessed (including with the use of biomarkers) at each visit, especially in patients with more frequent exacerbations, and that help be offered to those who wish to quit smoking. Although cotinine is a more accurate marker of tobacco smoke exposure than is the measure of exhaled CO, the latter can be used for this purpose, since it is inexpensive, it is measured instantaneously, and its values do not differ significantly between smokers with COPD and those without.^(^ 14 ^)^ A study conducted in the United Kingdom revealed that only 13% of smokers received prescriptions for smoking cessation treatment, although those with COPD were particularly likely to be prescribed such treatment.^(^ 15 ^)^ In addition, another study by the same group^(^ 16 ^)^ revealed a high (17%) prevalence of exposure to environmental tobacco smoke in asthma patients, and such exposure is known to be associated with exacerbations.^(^ 8 ^)^ Although studies have suggested that, with the aid of medications, patients with chronic disease have rates of cessation similar to those of patients without chronic disease,^(^ 11 ^)^ it is possible that patients with chronic lung disease who continue smoking have more difficulty in quitting smoking, requiring greater support as compared with smokers without such comorbidities, including greater screening for psychological morbidities associated with smoking,^(^ 17 ^)^ which can influence cessation success.
